# Factors associated with sinus bradycardia during crizotinib treatment: a retrospective analysis of two large‐scale multinational trials (PROFILE 1005 and 1007)

**DOI:** 10.1002/cam4.622

**Published:** 2016-01-28

**Authors:** Sai‐Hong Ignatius Ou, Yiyun Tang, Anna Polli, Keith D. Wilner, Patrick Schnell

**Affiliations:** ^1^ Chao Family Comprehensive Cancer CenterUniversity of California Irvine School of MedicineIrvineCalifornia; ^2^ Pfizer OncologyLa JollaCalifornia; ^3^ Pfizer OncologyMilanItaly; ^4^ Pfizer OncologyNew York CityNew York

**Keywords:** Anaplastic lymphoma kinase, blood pressure, bradycardia, crizotinib, heart rate, nonsmall cell lung cancer

## Abstract

Decreases in heart rate (HR) have been described in patients receiving crizotinib. We performed a large retrospective analysis of HR changes during crizotinib therapy. HRs from vital‐sign data for patients with anaplastic lymphoma kinase (*ALK*)‐positive nonsmall cell lung cancer enrolled in PROFILE 1005 and the crizotinib arm of PROFILE 1007 were analyzed. Sinus bradycardia (SB) was defined as HR <60 beats per minute (bpm). Magnitude and timing of HR changes were assessed. Potential risk factors for SB were investigated by logistic regression analysis. Progression‐free survival (PFS) was evaluated according to HR decrease by <20 versus ≥20 bpm within the first 50 days of starting treatment. For the 1053 patients analyzed, the mean maximum postbaseline HR decrease was 25 bpm (standard deviation 15.8). Overall, 441 patients (41.9%) had at least one episode of postbaseline SB. The mean precrizotinib treatment HR was significantly lower among patients with versus without postbaseline SB (82.2 bpm vs. 92.6 bpm). The likelihood of experiencing SB was statistically significantly higher among patients with a precrizotinib treatment HR <70 bpm. PFS was comparable among patients with or without HR decrease of ≥20 bpm within the first 50 days of starting crizotinib. Decrease in HR is very common among patients on crizotinib. The likelihood of experiencing SB was statistically significantly higher among patients with a precrizotinib treatment HR <70 bpm. This is the first large‐scale report investigating the association between treatment with a tyrosine kinase inhibitor and the development of bradycardia. HRs should be closely monitored during crizotinib treatment.

## Introduction

Crizotinib is a multitargeted tyrosine kinase inhibitor that inhibits anaplastic lymphoma kinase (ALK), ROS1, and mesenchymal‐epithelial transition (MET) receptors, and has demonstrated significant improvement in progression‐free survival (PFS) over chemotherapy as both first‐line and second‐line treatment of patients with *ALK*‐rearranged advanced nonsmall cell lung cancer (NSCLC) 1, 2. Crizotinib is generally well tolerated with visual effects, gastrointestinal disturbance, and peripheral edema noted as common side effects. Decreases in heart rate (HR) have also been observed with crizotinib 3, 4, and sinus bradycardia (SB) is listed as a warning in the product information 5. In order to characterize the HR changes associated with crizotinib treatment, we performed a large‐scale retrospective analysis of the characteristics of HR changes during crizotinib treatment in two multinational clinical trials and identified potential factors that may predispose patients to SB.

## Patients and Methods

### Patients and procedures

Patients with *ALK*‐rearranged advanced NSCLC enrolled in the single‐arm crizotinib trial PROFILE 1005 (NCT00932451) or in the crizotinib arm of the phase III randomized trial PROFILE 1007 (NCT00932893) were included in this retrospective analysis. The eligibility criteria for both PROFILE 1005 and PROFILE 1007 have been published elsewhere 2, 6. Briefly, patients with *ALK*‐positive advanced NSCLC were identified by break‐apart fluorescence in situ hybridization, except for some patients in PROFILE 1005 who were identified through immunohistochemistry or reverse transcription polymerase chain reaction. PROFILE 1005 allowed patients with Eastern Cooperative Oncology Group (ECOG) performance status (PS) 0–3, while PROFILE 1007 allowed those with ECOG PS 0–2 only. All patients had received prior treatment for their advanced disease and were treated with a starting dose of crizotinib of 250 mg orally twice daily. Concomitant medications that are strong inducers and/or inhibitors of CYP3A4 were not allowed. Each planned treatment cycle was 21 days in duration and computed tomography scans were to be performed every 6 weeks. Common Terminology Criteria for Adverse Events (CTCAE) version 4.0 were used to grade adverse events and Response Evaluation Criteria in Solid Tumors version 1.1 was used in the evaluation of tumor response. The data cut‐off dates were 15 February 2012 for PROFILE 1005 and 30 March 2012 for PROFILE 1007. All patients in PROFILE 1005 and 1007 gave written, informed consent to participate in the studies, which were approved by the relevant institutional review board.

### HR changes and blood pressure monitoring

Vital signs including HR and BP were recorded at the beginning of each treatment cycle. As specified in both protocols, patients with HR <40 bpm were required to suspend treatment with crizotinib until HR was ≥40 bpm. The HR prior to the first dose of crizotinib (pretreatment), lowest HR recorded postbaseline, magnitude of HR changes, and time to the lowest postbaseline HR and to within 5 bpm of the lowest recorded postbaseline HR were examined. Paired recordings of HR and systolic BP (SBP) as well as diastolic BP (DBP) during crizotinib treatment were also analyzed.

### Concomitant medications

Information on concomitant antihypertensive medications, beta‐blockers, and nondihydropyridine calcium channel blockers (CCBs), and on metoclopramide 7, which have been associated with bradycardia, was collected on the case report form. Concomitant medication was defined as a medication administered during the study irrespective of dosing or duration of administration.

### Statistical methods

Only patients with a recorded pretreatment HR and at least one postbaseline HR measurement were included in this retrospective analysis (*n *=* *1053). Comparisons between patients who experienced postbaseline SB and those who did not were performed using the Pearson's *χ* ^2^ test for dichotomous variables and the Student's *t*‐test for continuous variables.

The following clinical factors that may affect the risk of developing SB were evaluated by logistic regression analysis: age (<65 years vs. ≥65 years), gender (male vs. female), race (Asian vs. non‐Asian), ECOG PS (0/1 vs. ≥2), smoking history (never smoker vs. former/current), pretreatment HR (<70 bpm vs. ≥70 bpm), use of any concomitant antihypertensive medications (excluding beta‐blockers and nondihydropyridine CCBs, which were analyzed separately; yes vs. no), beta‐blocker use (yes vs. no), nondihydropyridine CCB use (yes vs. no), and metoclopramide use (yes vs. no).

PFS was evaluated in the subset of patients who had a HR decrease ≥20 bpm within the first 50 days of starting crizotinib treatment and in the subset of patients who did not. In this analysis, day 50 was taken as time zero for PFS, which was analyzed using the Kaplan–Meier method. The median event time and the corresponding two‐sided 95% confidence intervals (CIs; Brookmeyer–Crowley method) were provided. All analyses were performed with SAS statistical software, version 9.2 (SAS Institute, Cary, NC, USA).

## Results

### Study population

Of 1106 patients with *ALK*‐positive NSCLC treated with crizotinib across PROFILE 1005 and PROFILE 1007, a total of 1053 (84% from PROFILE 1005 and 16% from PROFILE 1007) had both baseline and postbaseline HRs recorded and were included in this retrospective analysis. 441 (41.9%) had at least one recorded postbaseline HR <60 bpm. Patients who experienced postbaseline SB were more likely to be older (mean age 54.0 years vs. 50.7 years; *P*<0.0001), to be non‐Asian (*P *=* *0.039), to have good PS (0/1; *P *<* *0.017), and to have used beta‐blockers (*P *=* *0.002). The likelihood of developing SB was not affected by gender, smoking status, or use of any antihypertensive medication (excluding beta‐blockers and nondihydropyridine CCBs; Table 1).

**Table 1:** Clinical and baseline characteristics of total study population

		Postbaseline HR		
	Total population (*n* = 1053)	<60 bpm (*n* = 441)	≥60 bpm (*n* = 612)	*P*‐value
Mean age, years (SD)	52.1 (12.3)	54.0 (12.4)	50.7 (12.0)	< 0.0001^a^
Median age, years (range)	52.0 (19–83)	55.0 (22–83)	50.0 (19–82)	
Age, years (SD)				
<65	887 (84.2)	354 (80.3)	533 (87.1)	0.003^b^
≥65	166 (15.8)	87 (19.7)	79 (12.9)	
Sex				
Male	453 (43.0)	195 (44.2)	258 (42.2)	0.505^b^
Female	600 (57.0)	246 (55.8)	354 (57.8)	
Race				
Asian	469 (44.5)	180 (40.8)	289 (47.2)	0.039^b^
Non‐Asian	584 (55.5)	261 (59.2)	323 (52.8)	
ECOG performance status				
0–1	899 (85.4)	390 (88.4)	509 (83.2)	0.017^b^
≥2	154 (14.6)	51 (11.6)	103 (16.8)	
Smoking status				
Never smoker	691 (65.6)	287 (65.1)	404 (66.0)	0.717^b^
Former smoker	324 (30.8)	140 (31.7)	184 (30.1)	
Current smoker	38 (3.6)	14 (3.2)	24 (3.9)	
Any use of antihypertensives^c^				
Yes	349 (33.1)	148 (33.6)	201 (32.8)	0.807^b^
No	704 (66.9)	293 (66.4)	411 (67.2)	
Use of beta‐blockers				
Yes	128 (12.2)	70 (15.9)	58 (9.5)	0.002^b^
No	925 (87.8)	371 (84.1)	554 (90.5)	
Use of nondihydropyridine CCB				
Yes	14 (1.3)	8 (1.8)	6 (<1.0)	0.244^b^
No	1039 (98.7)	433 (98.2)	606 (99.0)	
Use of metoclopramide				
Yes	292 (27.7)	123 (27.9)	169 (27.6)	0.921^b^
No	761 (72.3)	318 (72.1)	443 (72.4)	

bpm, beats per minute; CCB, calcium channel blocker; ECOG, Eastern Cooperative Oncology Group; HR, heart rate; SD, standard deviation.

a *t*‐test for comparison of means.

b *χ* ^2^ test of general association.

c Excluding beta‐blockers and nondihydropyridine CCBs.

### Magnitude of HR changes and time to lowest postbaseline HR recorded

The mean pretreatment HR for all patients (*n *=* *1053) was 88.3 bpm (median 86.0 bpm; Table 2). The mean pretreatment HR for the 441 patients with SB was 82.2 bpm, which was significantly lower than the mean pretreatment HR of 92.6 bpm for the 612 patients who did not have SB (*P*<0.0001). The mean maximum change in HR for all patients was a decrease of 25.0 bpm (range −85 bpm to +27 bpm). The mean maximum decrease in HR was 30.0 bpm for the 441 patients with SB, which was significantly greater than the mean decrease of 21.4 bpm for the 612 patients without SB (*P*<0.0001). The mean time to the lowest postbaseline HR recorded was significantly longer among the 441 patients with SB (20.2 weeks) compared with the 612 patients without SB (12.0 weeks; *P*<0.0001). A similar significant difference was observed when the same comparison was performed using the mean time to within 5 bpm of the lowest recorded postbaseline HR (14.2 weeks for patients with SB vs. 8.8 weeks for patients without SB; *P*<0.0001).

**Table 2:** Magnitude of HR changes

	Total population (*n* = 1053)	Postbaseline HR	
		<60 bpm (*n* = 441)	≥60 bpm (*n* = 612)
Magnitude of HR change, bpm			
Mean baseline pretreatment HR (SD)	88.3 (16.1)	82.2 (14.2)^a^	92.6 (15.9)^a^
Median baseline pretreatment HR (range)	86.0 (43.0–145.0)	80.0 (43.0–143.0)	91.0 (54.0–145.0)
Mean decrease in HR from baseline (SD)	25.0 (15.8)	30.0 (13.8)^a^	21.4 (16.1)^a^
Median decrease in HR from baseline (range)	24.0 (−27.0–85.0)	28.0 (1.0–85.0)	20.0 (−27.0–67.0)
Time to HR change, weeks			
Mean time to the lowest HR recorded (SD)	15.4 (14.1)	20.2 (15.5)^a^	12.0 (11.8)^a^
Median time to the lowest HR recorded (range)	9.6 (0.7–75.4)	15.1 (1.7–71.1)	7.7 (0.7–75.4)
Mean time to within 5 bpm of the lowest HR recorded (SD)	11.0 (10.4)	14.2 (11.3)^a^	8.8 (9.0)^a^
Median time to within 5 bpm of the lowest HR recorded (range)	6.4 (0.7–72.1)	9.4 (1.7–63.1)	6.1 (0.7–72.1)

bpm, beats per minute; HR, heart rate; SD, standard deviation.

a *P*<0.0001 (*t*‐test for comparison of means).

Among the 441 patients with postbaseline SB (and based on a total of 5645 measurements), only a minority (*n *=* *26; 5.9%) had a lowest HR <45 bpm, among whom the mean postbaseline HR was 54.9 bpm (standard deviation [SD], 11.5). A minority of patients (*n *=* *83; 18.8%) had a lowest HR between 45 and 49 bpm, among whom the mean postbaseline HR was 58.5 bpm (SD, 9.5), while a majority (*n *=* *332; 75.3%) had a lowest HR between 50 and 59 bpm, among whom the mean postbaseline HR was 66.0 bpm (SD, 10.2). At the time of data cut‐off, 65 of the 1106 crizotinib‐treated patients (5.9%) reported SB as an adverse event: 83% grade 1, 14% grade 2, and 3% grade 3.

### Duration of crizotinib treatment

The mean duration of crizotinib treatment for all patients (*n *=* *1053) was 29.7 weeks (range, 0.6–108.3 weeks). The mean duration of crizotinib treatment among 441 patients with postbaseline SB was significantly longer than that of the 612 patients without postbaseline SB (38.9 weeks vs. 23.1 weeks; *P*<0.0001).

### HR and BP

Across all recorded postbaseline BP measurements for all patients with simultaneous HR measurements (*n *=* *10,203 in 1053 patients), mean SBP and mean DBP were similar between patients grouped according to their lowest postbaseline HR: SBP, 119.5 and DBP, 67.4 (HR <45 bpm; *n *=* *26); SB, 114.9 and DBP, 66.7 (HR 45–49 bpm; *n *=* *83); SBP, 116.0 and DBP, 68.7 (HR 50–59 bpm; *n *=* *332); and SBP, 116.2 and DBP, 71.0 (HR ≥60 bpm; *n *=* *612; Table S1). Paired BP and HR recordings (*n *=* *4900) for patients who did not receive any relevant concomitant medication were available for 542 patients. In the presence versus absence of postbaseline SB, ≥40‐mmHg decreases in SBP from baseline and ≥30‐mmHg decreases in DBP were observed in 0.3% versus 0.6% (SBP) and 0.6% versus 0.7% (DBP) of recordings, respectively. Decreases in SBP and DBP were slightly more frequent in patients receiving concomitant beta‐blockers independent of the presence of postbaseline SB (paired BP and HR recordings [*n *=* *963]; Table 3).

**Table 3:** Effect of concomitant beta‐blocker use on BP^a^

	Number of paired readings (%)	
BP	HR <60 bpm	HR ≥60 bpm
No relevant medication		
≥40 mmHg ↓ SBP	14/4900 measurements (0.3)	31/4900 measurements (0.6)
≥30 mmHg ↓ DBP	27/4900 measurements (0.6)	36/4900 measurements (0.7)
Concomitant beta‐blockers		
≥40 mmHg ↓ SBP	9/963 measurements (0.9)	16/963 measurements (1.7)
≥30 mmHg ↓ DBP	17/963 measurements (1.8)	19/963 measurements (2.0)

bpm, beats per minute; DBP, diastolic blood pressure; HR, heart rate; SBP, systolic blood pressure.

a The analyses included only patients with both baseline and postbaseline HR records. All available HR and BP records obtained at the same time at any planned and unplanned visit during concomitant beta‐blocker use were analyzed. Once the concomitant medication was stopped, the records were excluded from the analysis.

### Clinical and baseline characteristics potentially associated with increased likelihood of SB

Multivariable logistic regression analysis was performed to assess if any clinical or baseline patient characteristics increased the likelihood of developing SB (Table 4). Patients who had a pretreatment HR <70 bpm had 5.0 times higher odds (95% CI, 2.9–8.2; *P*<0.0001) of developing SB than those who had a baseline HR ≥70 bpm. An ECOG PS of 0 or 1 was marginally associated with the development of SB during crizotinib treatment (*P *=* *0.0485). Beta‐blocker use was not associated with an increased risk of SB in a statistically significant manner, nor were age, gender, smoking status, race, or use of antihypertensive medications (excluding beta‐blockers and nondihydropyridine CCBs), nondihydropyridine CCBs, or metoclopramide, controlling for all other factors in the analysis.

**Table 4:** Potential association between clinical and baseline characteristics and the risk of developing SB^a^

	Odds ratio^b^ (95% CI)	*P*‐value^c^
Baseline clinical and patient characteristics		
Pretreatment HR (<70 bpm vs. ≥70 bpm)	4.929 (2.946–8.245)	< 0.0001
Age (≥65 years vs. <65 years)	1.434 (0.997–2.064)	0.0521
Gender (male vs. female)	1.154 (0.882–1.510)	0.2973
Smoking status (current/former vs. never smoker)	1.001 (0.757–1.323)	0.9954
Race (Asian vs. non‐Asian)	0.879 (0.671–1.151)	0.3488
ECOG PS (≥2 vs. 0/1)	0.686 (0.471–0.998)	0.0485
Concomitant medications		
Beta‐blocker use (yes vs. no)	1.390 (0.916–2.110)	0.1221
Nondihydropyridine CCB use (yes vs. no)	1.465 (0.470–4.569)	0.5109
Metoclopramide use (yes vs. no)	1.203 (0.894–1.618)	0.2232
General antihypertensive use^d^ (yes vs. no)	0.836 (0.629–1.111)	0.2174

bpm, beats per minute; CCB, calcium channel blocker; CI, confidence interval; ECOG PS, Eastern Cooperative Oncology Group performance status; HR, heart rate; SB, sinus bradycardia.

a SB is defined as any HR <60 bpm while receiving crizotinib treatment.

b Odds ratio >1 indicates higher odds of developing SB in the first category, and odds ratio <1 indicates higher odds in the second category.

c Wald *χ* ^2^ test.

d Excluding beta‐blockers and nondihydropyridine CCBs.

### PFS outcome according to HR decrease (<20 bpm vs. ≥20 bpm)

**Figure 1:**
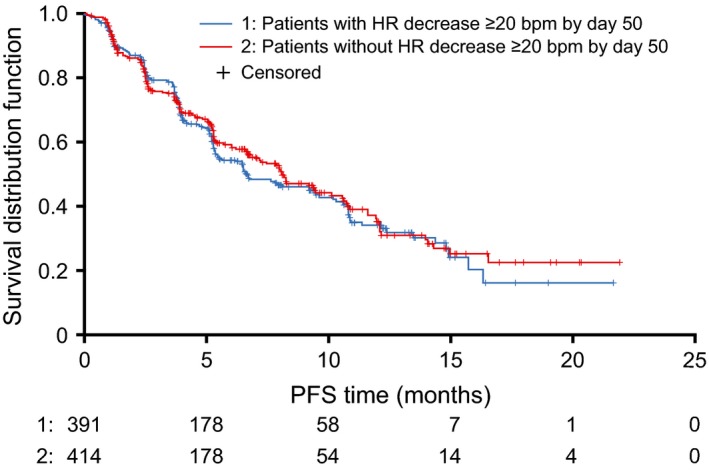
PFS by presence/absence of HR decrease ≥20 bpm by day 50. Day 50 was taken as time zero for PFS. bpm, beats per minute; HR, heart rate; PFS, progression‐free survival.

We investigated whether the PFS of patients who experienced a postbaseline HR decrease of ≥20 bpm within the first 50 days of starting crizotinib treatment differed from the PFS of those who did not experience such a postbaseline HR decrease. The 50 days window corresponds to the start of cycle 3 (day 43) plus a 7 day allowance to ensure that at least two postbaseline HR recordings were available. Patients who had PFS events (or were censored) within 50 days of starting crizotinib treatment were excluded from this analysis as they may not have had the opportunity to develop SB. In all, 805 patients were included in the PFS analysis. There were 391 patients (48.6%) with a HR decrease ≥20 bpm within the first 50 days of starting crizotinib treatment and 414 patients (51.4%) without a HR decrease ≥20 bpm within the first 50 days of starting crizotinib treatment. The median PFS for patients in the subgroup with a HR decrease ≥20 bpm was 6.7 months (95% CI, 5.5–9.5) and 8.1 months (95% CI, 6.9–10.0) among patients without a HR decrease of ≥20 bpm (Fig. 1).

## Discussion

In this large‐scale retrospective analysis of 1053 patients with *ALK*‐positive NSCLC, we showed that decreases in HR are common with the use of crizotinib, with a mean observed decrease of approximately 25 bpm among all evaluated patients. Additionally, 42% of evaluated patients had at least one episode of postbaseline HR of <60 bpm. The vast majority of these patients (75.3%) had a lowest recorded HR of between 50 and 59 bpm, with a mean postbaseline HR of 66 bpm. It is important to note that HR is dynamic and that even in the group of patients with the lowest recorded HR (<45 bpm), HR likely did not remain in this range for sustained periods, as suggested by the mean overall HR of 54.9 bpm in this group of patients. Postbaseline BP values obtained from 10,203 vital sign measurements taken among 1053 patients indicated that neither SBP nor DBP showed systematic changes as an apparent function of HR.

Importantly, the pretreatment HR of patients with postbaseline SB was significantly lower than in patients without postbaseline SB. Indeed, pretreatment HR <70 bpm was the strongest predictor of the development of SB by logistic regression analysis, with an observed five‐fold increase in the likelihood of developing SB during crizotinib treatment compared with patients with a pretreatment HR of ≥70 bpm.

While 40% of patients not treated with beta‐blockers developed SB (371 of 925), 55% of patients treated with beta‐blockers developed SB (70 of 128). This suggests that the concomitant use of beta‐blockers increases the risk of developing SB during crizotinib treatment, although this association did not reach statistical significance in the multivariable logistic regression analysis. For this reason, it is important for physicians to review medications that patients are taking before starting crizotinib and to consider alternatives to beta‐blockers as medically appropriate. Use of antihypertensives (excluding nondihydropyridine CCBs and beta‐blockers) was shown not to be predictive of changes in HR, while the available data on nondihydropyridine CCBs and on metoclopramide need to be interpreted with caution due to the small number of patients treated (with CCBs) and the high likelihood of intermittent use (of metoclopramide). In view of these limitations, physicians should continue to use caution when administering medications causing SB in patients treated with crizotinib.

In this analysis, another predictive factor for developing SB while on crizotinib was baseline ECOG PS 0 or 1, whereas age, gender, smoking status, and race were not predictive for SB during crizotinib treatment (by multivariable logistic regression analysis). The apparent association between ECOG PS and SB may be related to factors such as treatment exposure as patients with a poorer ECOG PS tend to receive treatment for shorter periods and may experience more dose reductions/interruptions.

The proportion of patients with SB was higher in this retrospective analysis of measured HR than that described in the product label of crizotinib 5, because labeling information is based on reported adverse events. The vast majority of patients with SB in this retrospective analysis were asymptomatic as evidenced by the low number of reported adverse events (grade ≥2) of bradycardia. Additionally, among the 1106 patients with *ALK*‐positive NSCLC treated with crizotinib across PROFILE 1005 and PROFILE 1007, only nine (1%) had a crizotinib dosing modification (dosing interruption and/or dose reduction) due to an adverse event of SB. In contrast to the grading of PR prolongation or QTc prolongation, which is based in part on duration as measured in milliseconds, CTCAE criteria (version 4.0) for grading SB are not based on the actual recorded HR, but rather on symptomatology and the need for medical intervention. Grade 1 SB is defined as a HR of <60 bpm that is “asymptomatic, interventions not indicated.” While it is generally accepted in the cardiology literature that HR <60 bpm is considered SB 8, physicians may not report an HR of 50–59 bpm as an adverse event if not symptomatic (grade 1).

This is the first large‐scale report investigating the association between treatment with a tyrosine kinase inhibitor and the development of bradycardia. The exact mechanism by which crizotinib affects HR remains unknown. There are now emerging data that another ALK inhibitor, ceritinib, can cause HR decreases and SB 9. Hence bradycardia, commonly observed during crizotinib treatment, may be a class effect of ALK inhibitors. However, both drugs can affect other molecular targets; crizotinib also inhibits ROS1 and MET tyrosine kinases, and ceritinib also inhibits ROS1 tyrosine kinase. Overall, HR should be closely monitored in ongoing trials involving these medications.

## Conflict of Interest

Sai‐Hong Ignatius Ou has received honoraria from Pfizer and Roche, and research funding from Pfizer, Roche, and AstraZeneca. He has served in a consultancy/advisory role for Pfizer and Roche, and has attended advisory boards on behalf of Pfizer, Roche, and Celgene. Yiyun Tang, Anna Polli, Keith D. Wilner, and Patrick Schnell are all Pfizer employees and own Pfizer stock.

## Supporting information


**Table S1.** SBP and DBP among patients grouped according to range of lowest postbaseline HRs^a^.
