# Supplementary material for: Factors associated with sinus bradycardia during crizotinib treatment: a retrospective analysis of two large‐scale multinational trials (PROFILE 1005 and 1007)
Source: Cancer Med. 2016 Jan 28;5(4):617–22. doi: 10.1002/cam4.622 (PMC4831279; doi:10.1002/cam4.622)
Supplement: Supplementary file 1 — Table S1. SBP and DBP among patients grouped according to range of lowest postbaseline HRsa. [file CAM4-5-617-s001.docx]

**Supplementary Table 1.** SBP and DBP Among Patients Grouped According to Range of Lowest Post-Baseline HRs^a^

|  | Range of Lowest HRs Recorded | | | |
| --- | --- | --- | --- | --- |
|  | <45 bpm | 45-49 bpm | 50-59 bpm | ≥60 bpm |
| Number of patients (%) | 26 (2.5) | 83 (7.9) | 332 (31.5) | 612 (58.1) |
| Number (%) of BP recordings by HR group^b^ | 404 (4.0) | 1,126 (11.0) | 4,115 (40.3) | 4,558 (44.7) |
| Mean SBP (SD^c^) | 119.5 (16.4) | 114.9 (15.4) | 116.0 (14.9) | 116.2 (14.0) |
| Median SBP (range) | 118 (82-179) | 113 (60-185) | 115 (73-184) | 116 (70-184) |
| Mean DBP (SD^c^) | 67.4 (10.0) | 66.7 (9.3) | 68.7 (10.0) | 71.0 (9.7) |
| Median DBP (range) | 67 (40-97) | 67 (36-99) | 69 (31-123) | 70 (35-108) |

Abbreviations: bpm, beats per minute; BP, blood pressure; DBP, diastolic blood pressure, HR, heart rate; SBP, systolic blood pressure; SD, standard deviation.

^a^ The analyses included only patients with both baseline and post-baseline HR records. All available HR and blood pressure records obtained at the same time at any planned and unplanned visits were analyzed.

^b^ Number of BP recordings with simultaneously recorded HR falling into one of the four indicated categories.

^c^ Calculated based on the total variation, including between-subject variation and within-subject variation.
